# Visual Outcomes and Complication Profiles of Phacoemulsification in Diabetic Versus Non-diabetic Patients

**DOI:** 10.7759/cureus.101827

**Published:** 2026-01-19

**Authors:** Nabanita Jena, Sudeep Saran, Aditi Mishra, Shivam Agarwal, Niraj Lodha, Hairya Ajaykumar Lakhani

**Affiliations:** 1 Department of Medical Surgical Nursing, Mother Theresa Postgraduate and Research Institute of Health Sciences, Affiliated to Pondicherry University, Puducherry, IND; 2 Department of Medicine, Saran Hospital and Institute of Paramedical Sciences, Bareilly, IND; 3 Department of Ophthalmology, Nandkumar Singh Chouhan Government Medical College (NSCGMC), Khandwa, IND; 4 School of Health Sciences, Chhatrapati Shahu Ji Maharaj University, Kanpur, IND; 5 Department of Internal Medicine, Lodha Hospital and Research Centre, Pali, IND; 6 Department of Internal Medicine, SBKS Medical Institute and Research Center, Vadodara, IND

**Keywords:** cataract surgery, diabetes mellitus, macular edema, phacoemulsification, visual outcomes

## Abstract

Diabetic patients undergoing phacoemulsification for cataracts face an elevated risk of intraoperative and postoperative complications due to systemic and ocular pathophysiological changes. This narrative review aims to systematically compare visual outcomes and complication profiles between diabetic and non-diabetic individuals following phacoemulsification, while identifying current best practices and future research directions. With rising global diabetes prevalence, cataract surgery in diabetic patients is increasingly common. Understanding the interaction between diabetes-related ocular changes and phacoemulsification outcomes is vital for improving surgical planning, reducing complications, and enhancing patient satisfaction. A comprehensive literature search was conducted across PubMed, Scopus, and Web of Science using Medical Subject Headings (MeSH) and free-text terms. Eligible studies from 2020 to 2025 were selected based on predefined criteria. Data were narratively synthesized across themes, including intraoperative challenges, visual acuity outcomes, macular health, adjunctive therapies, and long-term prognosis. Diabetic patients exhibit higher risks of poor pupil dilation, capsular fragility, macular edema, and slower visual recovery. However, with good glycemic control, preoperative retinal evaluation, and adjunctive therapies, outcomes can closely approximate those of non-diabetics. Tailored perioperative management and interdisciplinary care models are critical for optimizing outcomes in diabetic cataract surgery. Future studies should focus on stratified risk assessment and long-term visual prognosis.

## Introduction and background

Background on cataracts and phacoemulsification

Cataracts have been ranked among the most common causes of blindness in the world, and they have made a large share of the burden of preventable blindness [1]. With the aging population, particularly in developing countries, the rate and prevalence of blindness due to cataracts are likely to increase. Phacoemulsification and cataract surgery in general are considered one of the most effective, safe, and frequently undertaken ophthalmic procedures in the restoration of vision in patients with lens opacification [1].

Phacoemulsification is one of the major innovations in cataract surgery, which has overtaken the earlier methods like extracapsular and intracapsular cataract extraction. This technique makes use of ultrasonic energy to emulsify the opacified lens, then removes it by aspiration and replaces it with an intraocular lens (IOL), resulting in smaller incisions, rapid healing, and predictable refractive results. Moreover, phacoemulsification offers better control over the procedure and minimal inflammation after the procedure, which enables faster visual rehabilitation and a short hospital stay [1].

The surgical outcomes have also been further optimized over the years through the development of technology such as better viscoelastic substances, foldable IOLs, and high-speed phacoemulsification machines. Such inventions have enabled safer surgeries even in sensitive cases like a surgery in the eyes with comorbidities like diabetes mellitus [2].

Diabetes and ocular health

Diabetes mellitus is a systemic illness that has far-reaching consequences on ocular structures. Biochemical and metabolic alterations caused by hyperglycemia may also destroy several elements of the eye, such as the cornea, lens, retina, and optic nerve. Diabetic patients are reported to develop cataracts earlier and at a faster rate than their non-diabetic counterparts, and thus they pose a high-risk group of patients who require such surgery. The metabolic disruption of lens transparency by the build-up of sorbitol, advanced glycation end products, and oxidative stress all play roles in lens opacification with chronic hyperglycemia [3,4].

In addition, diabetic patients have a poor physiology of the ocular surface and anterior segment. Diabetic eyes are more susceptible to surgical trauma and complications due to abnormalities like lower corneal sensitivity, weak epithelial integrity, and poor endothelial functioning [2,5]. Specifically, there is reduced cell density and reduced regenerative ability of the corneal endothelium in diabetic patients, which may affect the clarity of the cornea and the healing process after surgery [6,7].

Phacoemulsification in diabetic patients, therefore, presents unique challenges. Issues such as poor pupil dilation, enhanced capsular fragility, and the possibility of worsening pre-existing diabetic retinopathy or macular edema should be controlled to bring the best results [8].

Prevalence of cataracts in diabetic populations

Several epidemiological studies posit that there is an increased prevalence of cataract formation in people with diabetes. The risk of cataract development among diabetic persons is almost two to five times higher than that of non-diabetic persons. Additionally, posterior subcapsular and cortical opacities are some forms of cataracts more prevalent in the eyes of diabetics [9].

The development of cataracts in such patients is bilateral, and their rate of progression is fast, hence the need for early surgery. Notably, in diabetic patients who have cataract surgery, there is usually co-occurring diabetic retinopathy or macular edema, which may affect visual prognosis [10].

Rationale for the review

Since there is an increasing prevalence of diabetes and cataracts worldwide, it is important to have an insight into the interplay between the two conditions to maximize patient management. Although phacoemulsification remains the most common method of performing this procedure, the effects of diabetes on the visual results and the pattern of complications are a matter of concern. Although most studies have reported the outcome of the diabetic eye, the evidence is heterogeneous, and the results are variable in terms of visual recovery, corneal endothelial cell loss, macular changes, and inflammatory responses. Also, perioperative management of diabetic patients, including the application of adjunctive treatment, e.g., intravitreal steroids or anti-vascular endothelial growth factor (VEGF), is still evolving. These complexities have to be considered in surgical planning and decision-making [11-13].

This narrative review seeks to give a detailed comparison of visual outcomes and complication profiles of diabetic patients and non-diabetic patients who have undergone phacoemulsification. Integrating the existing evidence and pointing out clinical implications, the review will aim to inform best practices and influence further studies within this significant field.

Current gaps in the literature

Although there have been several studies that have focused on a particular parameter, like endothelial alterations or macular thickness alterations after surgery, there are not many studies that have a comparative analysis of the holistic outcome, including intraoperative, short-term, and long-term outcomes. Furthermore, the timing and the nature of adjunctive treatment that would be most beneficial in diabetic eyes have limited agreement. The recent developments, like the combined application of intravitreal implants and phacoemulsification, are promising but still have to be validated [14].

## Review

Search strategy

To collect pertinent and exhaustive data on the visual results and complication profiles of phacoemulsification in diabetic and non-diabetic patients, a systematic literature search was conducted in various scientific databases. These were PubMed, Scopus, and Web of Science, which collectively provide comprehensive coverage of peer-reviewed medical and surgical literature. The search strategy aimed at covering both the modern evidence and the pioneer studies, especially the research published since 2020 up to 2025, by the latest trends in phacoemulsification technology and adjunctive treatment regimens.

The search utilized both Medical Subject Headings (MeSH) and free-text terms to increase retrieval sensitivity and specificity. Keywords included “phacoemulsification,” “diabetes mellitus,” “cataract surgery,” “visual outcomes,” “macular edema,” “corneal endothelium,” “non-diabetic,” “retinopathy,” and “complications.” Boolean operators such as “AND” and “OR” were employed to combine terms strategically. Manual cross-referencing of citations from identified articles was also conducted to ensure that potentially significant studies not captured by automated searches were included.

Inclusion and exclusion criteria

Studies had to be peer-reviewed, published in English, and categorized as an original study, systematic review, or meta-analysis to meet the inclusion criteria for this review. The results of phacoemulsification in adult patient populations were the focus of qualifying research, and patients with and without diabetes were clearly distinguished from one another. Studies with inadequately documented patient cohorts’ diabetes status and those conducted outside of clinical settings were not taken into consideration. Specifically, studies were excluded if diabetes status was not defined using established clinical diagnostic criteria, if laboratory confirmation, such as fasting plasma glucose or HbA1c levels, was absent or unreported, or if there was unclear differentiation between diabetic and non-diabetic groups in the study methodology. Additionally, studies were only allowed to be included if they included quantifiable and transparent outcome indicators, such as changes in macular thickness, corneal endothelial cell count, visual acuity, or rates of postoperative complications. Visual acuity outcomes in the included studies were reported using standard clinical assessment tools, including the Snellen chart and/or the logMAR scale, as specified in the original publications.

Research papers that had no empirical data, such as case reports, conference abstracts, editorials, or opinion pieces, were excluded from the study. Excluded were studies that did not distinguish between patients with and without diabetes, that employed surgical methods other than phacoemulsification or extracapsular cataract extraction, or that specifically targeted children. Additionally, animal models were excluded unless they provided useful mechanistic knowledge directly related to human practice.

Data extraction and synthesis

The manual extraction of the relevant information in the selected articles was done and systematized within predefined thematic categories. The data that were extracted were the objectives of the study, the population, the sample size, the presence of diabetic retinopathy (DR) or macular edema, the surgical method to be used, the type of IOL, whether the surgical method used adjunctive therapies, and the follow-up period. The outcome measures mainly concentrated on the postoperative visual acuity, corneal endothelial health, macular changes, and occurrence of surgical complications.

The study designs and outcome measures were very heterogeneous, so a narrative synthesis technique was deployed rather than a quantitative meta-analysis. This enabled it to determine clinical trends, discrepancies in results, and emerging agreement on various aspects of patient care. Findings have been displayed in the following sections of this review thematically.

Pathophysiological differences affecting surgical outcomes

Ocular Surface and Tear Film Changes in Diabetes

Diabetes mellitus brings a lot of changes in the ocular surface, and these changes have direct consequences in the postoperative recovery after phacoemulsification. Chronic hyperglycemia affects the stability of the tear film and integrity of the corneal epithelium, which causes higher rates of dry eye symptoms and a slower rate of epithelial healing. This is commonly explained by the decrease in corneal sensitivity, disruption of the lacrimal gland, and microvascular damage of the ocular innervation [15]. It has been established recently that diabetic patients have stronger postoperative inflammation and a lower rate of tear film recovery, which can cause temporary visual dysfunction and pain [16].

Pupillary dynamics are also notably altered in diabetic eyes. After phacoemulsification, the diabetic patient may show a poor pupillary reaction and a lower pupil diameter than the normal patient, which is evidence of autonomic dysfunction due to chronic hyperglycemia [12,16]. These may make the surgery more complicated and affect the visual rehabilitation in the early postoperative phase.

Diabetic Retinopathy and Macular Edema Risk

DR and the possible occurrence and worsening of diabetic macular edema (DME) are a big issue during and after phacoemulsification in diabetics. Post-operative inflammatory cascade can stimulate the rate of retinal microvascular leakage, causing new or exacerbated macular edema [17,18]. Research has established that surgical trauma may augment intraocular concentrations of VEGF, particularly in individuals with pre-existing non-proliferative diabetic retinopathy (NPDR) and further augment the likelihood of postoperative vision loss because of edema [4,19].

It is also difficult to view the fundus during surgery in diabetic white cataracts as a result of poor red reflex and greater lens opacity. A recent study compared three intraoperative fundus inspection methods and concluded that detection of the retinal pathology at an early stage and intraoperative evaluation of the retinal pathology are essential in preventing post-surgery visual deterioration [13].

To reduce the occurrence of these risks, intraocular adjunctive treatments like intravitreal dexamethasone or anti-VEGF agents are being increasingly used during and/or immediately after phacoemulsification in diabetic patients with DME. Research indicates that co-administration of these agents in cataract surgery enhances anatomical and functional results [3,14,20].

Poor Wound Healing and Corneal Endothelial Dysfunction

The health of the corneal endothelium is important to preserve postoperative clarity and limit edema. The endothelial cell density and pump activity are frequently diminished in diabetic cornea and make it more vulnerable to the effects of phacoemulsification [21-23]. This is due to endothelial susceptibility that is caused by chronic oxidative stress, deposition of advanced glycation end-products, and mitochondrial abnormalities in corneal tissues. An interesting study attributed increased endothelial cell loss in diabetic patients to elevated levels of oxidative stress after surgery, which underlines the importance of careful surgical treatment and sufficient protection during surgery [24,25].

The thickness of corneas and their dynamics after the surgery are different in diabetics. Spectral-domain optical coherence tomography (OCT) and specular microscopy revealed that the epithelial remodeling was protracted and the normal architecture was not recovered normally in diabetic corneas after surgery [10,21]. Such modifications are linked with poor visual recovery and temporary opacification of the cornea or corneal edema.

**Figure 1:**
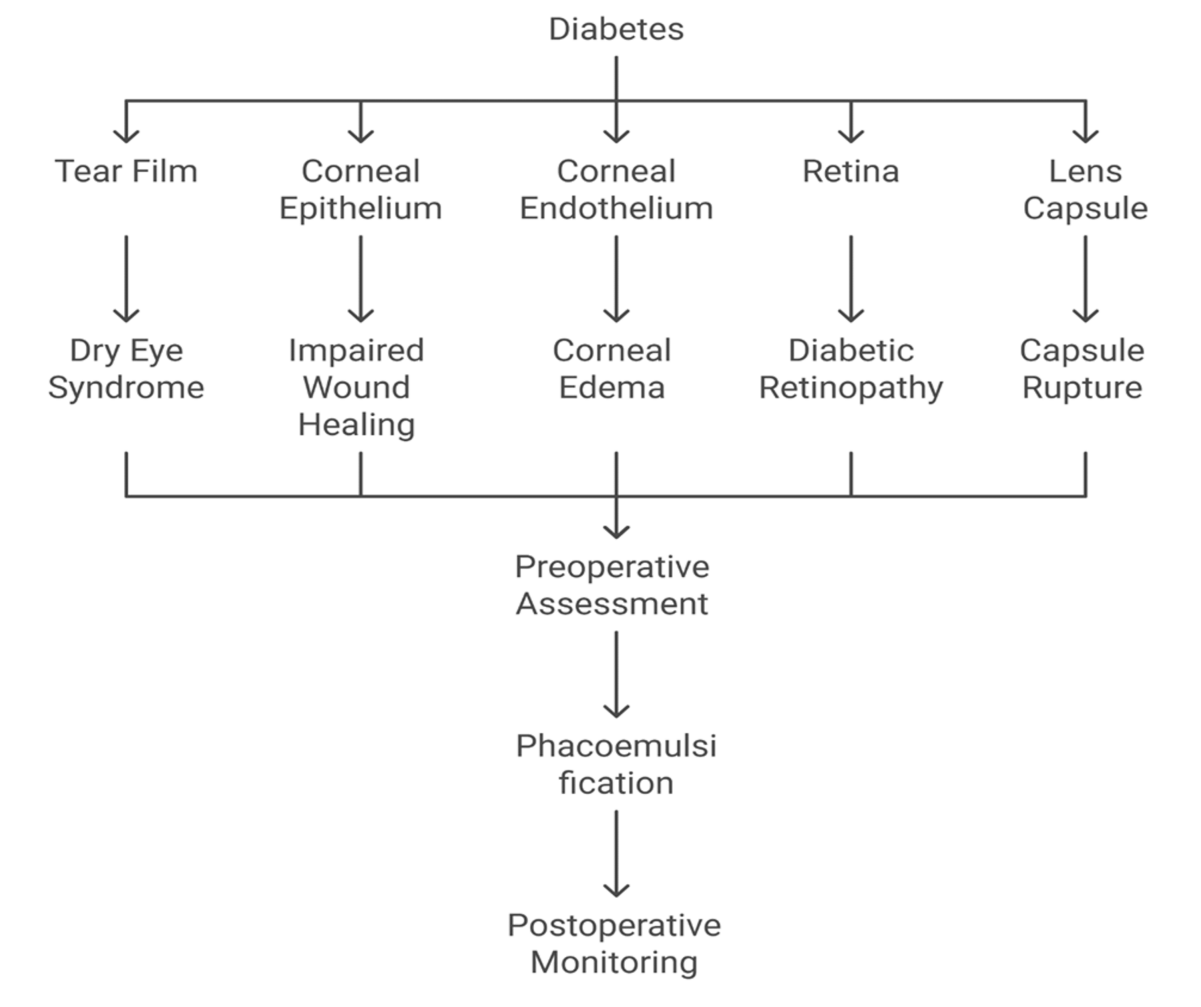
Pathophysiological Impact of Diabetes on Ocular Tissues Created by authors

Femtosecond laser-assisted phacoemulsification has been suggested to decrease the cumulative dissipated energy and endothelial cell loss. It has been shown that such an approach can be safer on diabetic corneas, providing more consistent postoperative results [11,23]. Figure 1 shows the multifactorial pathophysiological changes in diabetic eyes, such as corneal endothelial susceptibility, poor healing, and inflammation.

Visual outcomes after phacoemulsification

Postoperative Visual Acuity: Diabetic vs Non-Diabetic Patients

Short-term (1-3 months): Non-diabetic patients usually experience visual recovery following phacoemulsification quickly, and most of them record significant recoveries within weeks. Nevertheless, diabetic patients tend to have a less rapid response to recovery, particularly when there is retinopathy or subclinical macular edema. The results of the studies are that diabetic patients do gain vision at one to three months after surgery, but the amount of improvement is sometimes less than that of the non-diabetics [9,17,22]. In addition, even mild DR without evident macular involvement has been linked to poor visual recovery because of minor changes in retinal structure. The addition of topical ketorolac and systemic normalization of blood glucose has provided some success in enhancing the recovery rate and inhibiting subclinical inflammation [15].

Long-term (6 months-1 year): Over the long term, diabetic and non-diabetic groups tend to stabilize in visual outcomes, especially in those patients with good glycemic control and no or minimal retinopathy. Within six to twelve months after the surgery, visual acuity of well-managed diabetics is almost equal to that of non-diabetics, as long as there are no progressive retinal complications [5,26]. However, the risk of late complications, such as the development of DR and DME recurrence, is still increased in diabetics. Thus, postoperative monitoring and retinal follow-up are very important to maintain the visual gains [27-30]. A study observed that early postoperative or concomitant intravitreal treatment was able to preserve visual outcomes in the 12-month study period, particularly in patients with baseline macular pathology [31].

Influence of Diabetic Retinopathy Stage

The predictive value of the DR severity at the time of intervention is associated with the visual outcome. Patients who have no retinopathy or mild NPDR do well and have good postoperative gains. Nevertheless, individuals with moderate to severe NPDR or proliferative diabetic retinopathy (PDR) tend to have more complex and complicated courses, such as the ongoing macular edema, posterior segment inflammation, and slow visual recovery [9,14,28]. An observational study comparing visual development in eyes with various levels of DR showed that there was little difference in the visual outcomes between eyes with early-stage disease and no significant effect, but eyes with PDR had more heterogeneity in response and a higher incidence of further intervention after surgery [29]. The anti-VEGF prophylactic treatment of such cases has been demonstrated to stabilize retinal vasculature and to improve the prognosis of vision [30,32].

Impact of Glycemic Control (HbA1c Levels) on Visual Recovery

The level of HbA1c is a major determinant of surgical outcomes in diabetic patients, and preoperative glycemic control is a major determinant of surgical outcomes. High HbA1c is linked to high surgical risk, more postoperative complications, and less visual improvement. A lack of glycemic control worsens the endothelial dysfunction of the cornea, inflammation, and vascular permeability of the retina [18,24]. Patients with good control of diabetes (HbA1c 77), on the other hand, experience quicker healing, reduced complication rates, and improved long-term vision results. In a retrospective study, it was pointed out that the effect of strict glycemic control before surgery was positively correlated with corneal clarity and central macular thickness, which ensured improved vision after the operation [19].

**Figure 2:**
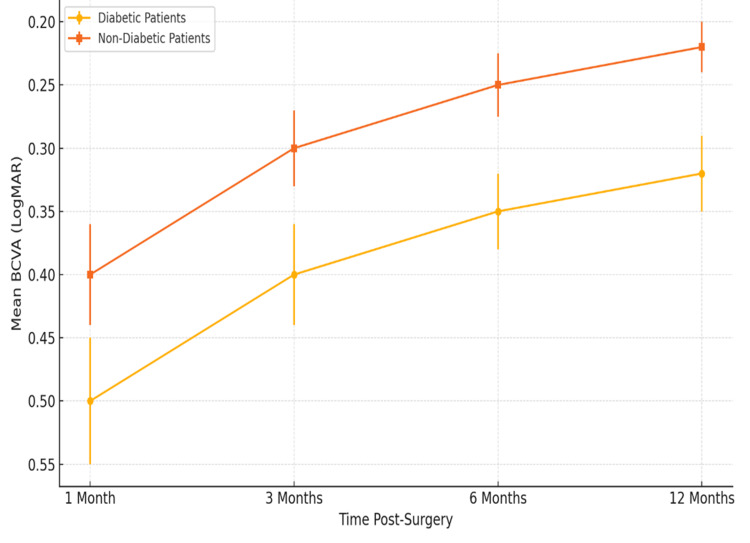
Trajectory of Postoperative Visual Acuity Recovery Created by authors BCVA: best-corrected visual acuity

Some authors support postponing elective cataract surgery in patients with uncontrolled diabetes until the metabolic systemic parameters are stable. The method reduces both intraoperative and postoperative complications and patient satisfaction [22,27]. Figure 2 indicates the variations in outcomes of visual acuity recovery trends in diabetic and non-diabetic patients within a 12-month follow-up period, as well as the impact of systemic control on ocular healing. The comparative trend in visual recovery between diabetic and non-diabetic patients at various postoperative timepoints is illustrated in Figure 2, based on data synthesized from Bassi et al. [9], Gao et al. [25], and Raimondi et al. [30].

Intraoperative and postoperative complication profiles

Intraoperative Challenges in Diabetic Eyes

Phacoemulsification in diabetic patients presents several unique intraoperative difficulties. Poor pupil dilation is one of the problems that is the result of autonomic neuropathy, which causes a lack of responsiveness in the iris musculature. The literature has always identified reduced intraoperative and baseline pupil size in diabetic patients compared with non-diabetics, making it difficult to visualize the surgical eye and causing more mechanical iris injury [32,33]. The other intraoperative issue is the high fragility of the capsule, which is explained by the modification of the structure of lens capsules because of glycation in diabetics. The anterior capsule of a diabetic patient can be found to be less elastic, and it is more likely that the radial tear or an unstable capsulorhexis can occur during the surgery [34]. Moreover, in diabetic patients, intraoperative evaluation of the retina becomes more difficult as the posterior segment is usually poorly visualized in dense or white cataracts [13].

Early Postoperative Complications

Compared to diabetic patients, enhanced inflammation is more frequent as one of the early postoperative complications. Hyperglycemia modifies the inflammatory pattern and the wound healing of the eye, raising the cellular response in the anterior chamber and protein exudation. It can also lead to posterior synechiae and high intraocular pressure due to this inflammation [35,36]. Macular edema is a major postoperative issue with DME and cystoid macular edema (CME). Surgical stress and inflammatory stress can worsen active or subclinical DME, especially in patients who have preexisting DR. Thickening of the macula (i.e., increased central macular thickness related to diabetic or CME) is more long-lasting in diabetic eyes, following otherwise successful surgeries [37-39]. In a comparative study, elevated levels of VEGF and impaired retinal vasculature were identified as factors increasing the risk of macular fluid after the surgery [4]. Posterior capsule opacification (PCO) also develops in diabetic patients earlier and more often. It is related to the enhanced growth of lens epithelial cells and uncontrolled response to wound healing in hyperglycemic conditions. According to clinical studies, diabetic patients present with the need for YAG capsulotomy earlier than non-diabetic patients [40].

Late Complications

With time, diabetic patients are prone to the development of DR, particularly in cases where the disease is not well-managed before surgery. Even though phacoemulsification is not directly related to retinopathy, the inflammatory environment that follows the surgery can fast-track the process. Another study indicated substantial advancement of NPDR after uncomplicated cataract surgery [41]. Another late effect is secondary glaucoma, which may be a result of chronic inflammation or steroid response, or compromise of the trabecular meshwork. It can also be linked with the fragments of lens material or retained viscoelastic agents in diabetic eyes [42]. Moreover, continuing CME in diabetic patients has been recorded months after surgery, and in some cases, a second intravitreal treatment is required [39,43]. In a prospective cohort study, diabetic patients who received cataract extraction exhibited more variability in recovery of visual acuity as the time progressed than non-diabetics, especially in the presence of pre-existing macular abnormalities [44].

Comparison summary: diabetics vs non-diabetics

Overall, diabetic patients are prone to intraoperative and postoperative complications more than non-diabetic patients. These are impaired pupillary dilation, weak capsules, more inflammation, a greater risk of PCO, worsening of macular edema, and an enhanced risk of progressive retinopathy or glaucoma. Such risks should be identified and handled to customize surgical approaches and maximize positive postoperative outcomes among diabetic patients [32,44]. Table 1 shows a combined comparison of intraoperative and postoperative trends of complications.

**Table 1:** Comparative Analysis of Intraoperative and Postoperative Complications in Diabetic vs Non-diabetic Patients Undergoing Phacoemulsification LECs: lens epithelial cells; VEGF: vascular endothelial growth factor; IOLs: intraocular lenses; OVDs: ophthalmic viscosurgical devices; OCT: optical coherence tomography; DR: diabetic retinopathy; IOP: intraocular pressure; NSAIDs: nonsteroidal anti-inflammatory drugs; MGD: meibomian gland dysfunction; YAG: yttrium–aluminum–garnet (laser)

Complication Type	Description of Diabetics	Frequency Compared to Non-diabetics	Clinical Significance	Management Strategies	Reference
Poor Pupil Dilation	Autonomic dysfunction leads to miosis	More common	Hinders visualization during surgery	Use of mechanical dilators, pre-op mydriatics	[12,32,33]
Capsular Fragility	Glycation affects capsule elasticity	Increased	Risk of radial tears	Gentle capsulorhexis, viscoelastics	[34,13]
Inflammation	Hyperglycemia enhances the inflammatory response	Higher severity	Anterior chamber reaction, synechiae	Prolonged steroids, NSAIDs	[35,36]
Macular Edema	Surgery induces VEGF release	More frequent	Causes vision blurring	Intravitreal anti-VEGF/steroids	[4,18,20,37]
Posterior Capsule Opacification	Early proliferation of LECs	More rapid onset	Need for YAG laser capsulotomy	Hydrophobic acrylic IOLs	[40,45]
Endothelial Cell Loss	Diabetics have compromised corneal endothelium	Significantly higher	Leads to corneal edema	Dispersive OVDs, low energy settings	[7,21,24]
Corneal Edema	Delayed resolution and prolonged remodeling	Longer-lasting	Blurred vision	Monitor with OCT, osmotic agents if needed	[21,25,43]
Retinopathy Progression	Post-op inflammation may worsen DR	Higher risk	Long-term visual decline	Close retinal follow-up, pre-op retinal treatment	[44,41]
Glaucoma	Steroid response, retained viscoelastic	Increased incidence	IOP rise	Monitor IOP, adjust meds	[42]
Visual Recovery Time	Slower recovery in the presence of DR or edema	Delayed compared to controls	Extended follow-up needed	Optimize glycemic control	[17,26,28]
Infection Risk	Poor wound healing in hyperglycemia	Marginally increased	Delayed epithelial healing	Perioperative antibiotics, glycemic control	[6]
Tear Film Instability	Dry eye symptoms are more common post-op	Greater incidence	Affects comfort and vision	Artificial tears treat MGD	[15,16,22]

Role of adjunctive therapies and surgical techniques

Use of Anti-VEGF Agents and Steroids

To reduce the development of macular edema and inflammatory complications, the administration of anti-VEGF agents (e.g., bevacizumab, conbercept), intravitreal corticosteroids (e.g., dexamethasone implants) has become prevalent. Several reports have supported the fact that the combined use of these agents during the phacoemulsification procedure helps in decreasing the central macular thickness and enhances the long-term visual outcomes of diabetic individuals with pre-existing or high-risk DME [45-47]. In NPDR, a 2021 randomized trial highlighted that prophylactic intravitreal steroid administration during the surgery better accounted for the anatomical and functional improvement of the patients [38]. Similarly, preoperatively and within the immediate postoperative period, anti-VEGF therapy was demonstrated to stabilize the circulation of the retina and to avoid CME [48].

Phacoemulsification Modifications for Diabetics

Because diabetic eyes are more fragile, it is advisable to make some changes in the surgical techniques. Utilization of smaller incisions and low-energy phacoemulsification settings is employed to minimize the corneal thermal and mechanical injuries. The viscoadaptive ophthalmic viscosurgical devices (OVDs) can be applied to protect the endothelium during the phacoemulsification [23,25]. The eight Chop method, whereby the nucleus is divided into smaller portions mechanically and subsequently emulsified, helps in the reduction of total dissipated energy and endothelial trauma, especially in diabetic eyes [26]. Choice of IOLs is also a factor that determines postoperative results. The use of hydrophobic acrylic lenses is recommended because of their reduced association with PCO development [49]. Multifocal IOLs are avoided by surgeons in use in diabetic patients with pathology of the retina because of the fear of their reduced contrast sensitivity and visual quality [50,51].

Role of Preoperative Retinal Evaluation and Management

Preoperative retinal evaluation is vital in diabetic patients. Subclinical macular edema is identified by OCT, and fluorescein angiography can be justified in the evaluation of ischemic alterations or neovascularization. The detection of DME or PDR before the cataract surgery allows the retina to be intervened on time. Preoperative retinal therapy involving pan-retinal photocoagulation or anti-VEGF injections can help patients whose macular pathology is significant to prevent the occurrence of vision loss after the surgery. It was demonstrated that patients who underwent such preoperative interventions were more likely to achieve visual recovery and fewer postoperative complications [48,49].

In one of the trials, it was shown that a combination of phacoemulsification with an injection of conbercept into the eye resulted in better visual acuity and decreased macular thickness in individuals with comorbid DME, which highlights the importance of treating the retina preemptively [35]. To sum up, a more personal approach consisting of a surgical and pharmacologic plan with the help of a thorough preoperative assessment, surgical accuracy, and anti-inflammatory management is necessary to achieve the best results in diabetic patients who undergo phacoemulsification [52,53]. Table 2 provides an overview of different adjunctive options and their clinical significance in diabetic patients who undergo phacoemulsification.

**Table 2:** Summary of Adjunctive Interventions and Their Clinical Impact in Diabetic Patients Undergoing Phacoemulsification VEGF: vascular endothelial growth factor, DME: diabetic macular edema, DR: diabetic retinopathy, NPDR: non-proliferative diabetic retinopathy, CME: cystoid macular edema, OCT: optical coherence tomography, PDR: proliferative diabetic retinopathy, NSAIDs: nonsteroidal anti-inflammatory drugs, IOLs: intraocular lenses, PCO: posterior capsule opacification, phaco: phacoemulsification

Intervention Type	Application Stage	Purpose	Outcome Improvement	Patient Subgroup Benefited	Reference
Anti-VEGF Injections	Pre-/Intra-/Post-op	Reduce macular edema and vascular permeability	Reduced central macular thickness	DME or high-risk DR	[20,30,35]
Intravitreal Steroids	Intraoperative	Anti-inflammatory and anti-edematous effects	Faster recovery, reduced edema	NPDR and CME patients	[14,38,39,47]
Glycemic Optimization	Preoperative	Reduces surgical risk and postoperative inflammation	Fewer complications	All diabetic patients	[18,24,54,55]
OCT Screening	Pre- and Post-op	Detect subclinical macular edema	Early intervention	Patients with mild NPDR	[5,30]
Pan-retinal Photocoagulation	Pre-op in PDR cases	Prevent neovascularization	Reduced DR progression	Proliferative DR	[48,49]
Femtosecond Laser-Assisted Surgery	Intraoperative	Precision, low energy use	Reduced endothelial loss	Corneal endothelial dysfunction	[11,23]
Eight-Chop Technique	Intraoperative	Minimize phaco energy	Lower endothelial damage	Fragile corneas	[26]
NSAIDs + Corticosteroids	Postoperative	Control inflammation	Shortened inflammation duration	All diabetic eyes	[15,36,56]
Monofocal Hydrophobic IOLs	Intraoperative	Reduce PCO risk	Lower incidence of PCO	All diabetic patients	[49,50]
Conbercept + Phaco	Intra/Post-op	Anti-VEGF impact on macular outcomes	Enhanced macular stability	Concurrent DME	[35,31]
Tear Film Management	Pre-/Post-op	Control dry eye and surface instability	Improved comfort and healing	All diabetics with ocular surface issues	[15,22]
Collaborative Care Models	Perioperative	Systemic-metabolic control, retina–cataract coordination	Reduced overall complication rate	High-risk diabetics with systemic comorbidities	[36,48,53]

Patient counseling and perioperative management

Importance of Glycemic Control Pre-Surgery

Preoperative glycemic control before any cataract surgery is a critical requirement to reduce complications and enhance positive surgical predictors in diabetic patients. Uncontrolled diabetes has been deemed a factor in prolonged wound recovery, prolonged postoperative inflammation, and susceptibility to complication development, including macular edema and retinopathy [54,55]. Literature proves that a high level of HbA1c is associated with poor corneal and retinal outcomes following phacoemulsification, and systemic metabolic stabilization before surgical intervention is necessary [18,24].

Patient Education and Realistic Expectations

Appropriate preoperative counseling is important in the process of setting expectations and offering informed consent. Diabetic patients should know that even though phacoemulsification usually results in better vision, this process can be restricted by existing macular disease or retinal ischemia. It is also vital to teach patients about possible adjunctive therapies, including intravitreal injections, and the risk of slower visual improvement than their non-diabetic counterparts [56]. Besides, systematic review and meta-analysis of the available evidence revealed the effectiveness of phacoemulsification combined with prophylactic anti-VEGF to mitigate the burden of DME and maintain vision in high-risk diabetic groups [56].

There should also be counseling of the patients on the potential complications, which include a prolonged macular edema, PCO, and secondary glaucoma. Patient education about these risks can instill improved adherence to post-operative follow-up and treatment. Furthermore, deliberation on the necessity of regular retinal assessment following cataract surgery facilitates the establishment of realistic expectations of maintaining long-term sight [41,43].

Postoperative Monitoring Protocols

Close attention to anterior segment inflammation, the condition of the macula, and intraocular pressure should be provided in the postoperative management of diabetic patients. OCT plays an essential role in the early diagnosis of macular edema that can lead to the early treatment and maintenance of vision [57]. Supervision must be done at least 3 to 6 months after the operation, particularly when the patient has a history of DME or moderate to severe NPDR. Researchers have reported that the rise in intraocular pressure and the alteration in the thickness of the macula are more common in diabetic patients and may be observed even after uncomplicated surgeries [58,59]. Therefore, diabetics have the advantage of special follow-up plans with retinal specialists, besides the cataract surgeon.

Key findings from recent literature

The recent comparative studies all show that phacoemulsification is beneficial to both diabetic and non-diabetic patients, but the former have more complications and slightly poorer visual results, at least in the short term. To give an example, a meta-analysis assessing the alterations of endothelial cells after phacoemulsification revealed that there was a significant endothelial cell loss and slower corneal recovery in diabetic patients than in non-diabetic controls [8,60,61]. A different meta-analysis evaluating the postoperative macular thickness concluded that diabetic patients are more likely to experience transient or persistent edema regardless of whether they exhibit overt retinopathy or not. Anti-VEGF therapy and corticosteroids were found to reduce this risk, but they were needed for select patients [56].

Visual recovery also varied based on retinopathy stage. Non-diabetic patients with no DR or mild non-proliferative changes showed very close results with non-diabetics. Conversely, patients with moderate to severe DR or macular edema did not perform as well, and this was irrespective of the best surgical technique [5,9,31]. Table 3 provides a comparative summary of the major studies that have assessed phacoemulsification outcomes of diabetics as compared to non-diabetics, depicting variations in visual recovery, macular response, and endothelial integrity.

**Table 3:** Comparison of Major Studies VEGF: vascular endothelial growth factor, DME: diabetic macular edema, phaco: phacoemulsification, post-op: postoperative

Study	Population	Intervention	Key Outcomes
Yang et al. [8]	14 studies (n > 1,000)	Phacoemulsification	Higher endothelial cell loss in diabetics
Azhan et al. [4]	60 patients	VEGF levels in tears	Higher VEGF in diabetics post-op
Bassi et al. [9]	75 diabetic eyes with DME	Phacoemulsification alone	Visual gain, but less than in non-DME eyes
Montrone et al. [3]	58 eyes	Dexamethasone implant + phaco	Improved macular outcomes
Wang et al. [35]	60 eyes	Conbercept + phaco	Better macular thickness control

Evidence gaps and methodological limitations

Although there is an increasing body of literature, most studies are limited in some way that influences their generalizability. Sample sizes tend to be small, and there is wide variation in surgical technique, duration of diabetes, and retinopathy grading, which makes comparisons hard. Less data also exists regarding the effect of newer surgical modalities (e.g., femtosecond-assisted phacoemulsification) and long-term outcomes beyond one year [11,23].

The other important disparity is that of stratification using glycemic control. The prospective assessment of visual and anatomical outcomes according to the HbA1c preoperative level is limited, although metabolic control is a recognized factor in the control of healing and inflammation [18,59]. Moreover, hyperglycemia plays a role in augmenting oxidative stress, mitochondrial dysfunction, and inflammation, which deteriorate ocular tissues. Inflammatory pathways for IL-6/STAT3 activation are also elevated in a hyperglycemic setting, and molecular evidence supports the postponement of elective cataract surgery until glycemic parameters have been sufficient [59].

Controversies and conflicting data

There are conflicting findings on the best time and the kind of adjunctive therapy. Whereas there are authors who recommend preoperative anti-VEGF injections to prevent macular edema, the latter do not show any significant advantage, unless active edema exists [45,46,56]. Likewise, controversy still exists regarding the cost-effectiveness and the need for intravitreal steroid implants in regular cataract surgery in diabetics without macular pathology. There is yet another controversy on whether cataract surgery hastens the progression of DR. Some evidence shows that there is a definite relationship, whereas others indicate that the noted progression is a natural process of the disease and is not surgery-induced [44,41].

Clinical implications and best practice guidelines

Tailored Protocols for Diabetic Patients

Given the increased risk profile in diabetic patients, perioperative management should follow individualized protocols. Improvement of glycemic status, blood pressure, and renal function before surgery is required. A uniform practice can include the preoperative OCT to exclude macular edema, selective steroid or anti-VEGF application, and altered surgical parameters to reduce endothelial damage [6,25,26]. It is suggested that intraoperative measures like application of dispersive viscoelastics and less phacoemulsification energy (e.g., the use of Eight-Chop or stop-and-chop techniques) can be used to minimize the trauma to damaged corneal endothelium [26]. The choice of high optical quality monofocal IOLs is still the norm among diabetics, especially in the presence of a suspicion of macular pathology.

Risk Stratification Tools

Risk stratification tools should be developed and deployed to identify diabetic patients at higher risk of suboptimal outcomes after undergoing phacoemulsification. Many factors ought to be put into consideration when risk is assessed. These factors are the levels of HbA1c in the patient, which characterizes the glycemic control; the duration of the disease, because the longer the disease, the more likely the patient is to have more advanced ocular complications; and the stage of DR at the time of the surgery. Also, the presence of DME, prior intravitreal anti-VEGF therapy, and baseline macular thickness using OCT are essential factors that can indicate the possible complexity of surgery and visual outcome.

Stratifying patients according to these parameters helps the ophthalmologists plan. This can include the use of intravitreal treatment as concomitant to cataract surgery in individuals with active macular disease, or a more aggressive postoperative follow-up schedule in high-risk patients. This individualized planning can minimize the risk of complications and increase long-term visual outcomes of diabetic patients who undergo phacoemulsification [30,48,52].

Recommendations for surgeons and ophthalmic teams

The effective management of diabetic patients who are going through a cataract surgery requires a multidisciplinary approach. Adoption of routine preoperative retinal assessment (ideally by retina specialists) is recommended by surgeons and the ophthalmic teams to be able to identify and treat any macular pathology or progression of the retinopathy before the surgery takes place. In addition, anti-inflammatory prophylaxis should not be limited to the common non-steroidal anti-inflammatory drug (NSAID) regimen, but adjuvant corticosteroids or anti-VEGF should be used as needed.

It is also important to recognize and treat postoperative inflammation and edema early, especially in the presence of a pre-existing diabetic eye disease. Education of the patients must be emphasized, in which they must know the warning symptoms of visual decline and the necessity of following up. Lastly, the complication rates can also be minimized by closely working with endocrinologists to establish metabolic control in the system before and after surgery. Use of a structured checklist and development of shared care models between cataract and retina services have been demonstrated to be of high value in producing positive outcomes in high-volume ophthalmic practices [36,48].

Limitations

Although this narrative review offers a fully detailed synthesis of the current evidence regarding the outcomes of phacoemulsification in diabetic and non-diabetic patients, a number of limitations should be mentioned. To start with, most of the studies available are observational or retrospective in nature, and there is a relative dearth of randomized controlled trials that specifically target the diabetic cohorts. This confines the potency of causal stipulations on the risk factors and interventions.

**Figure 3:**
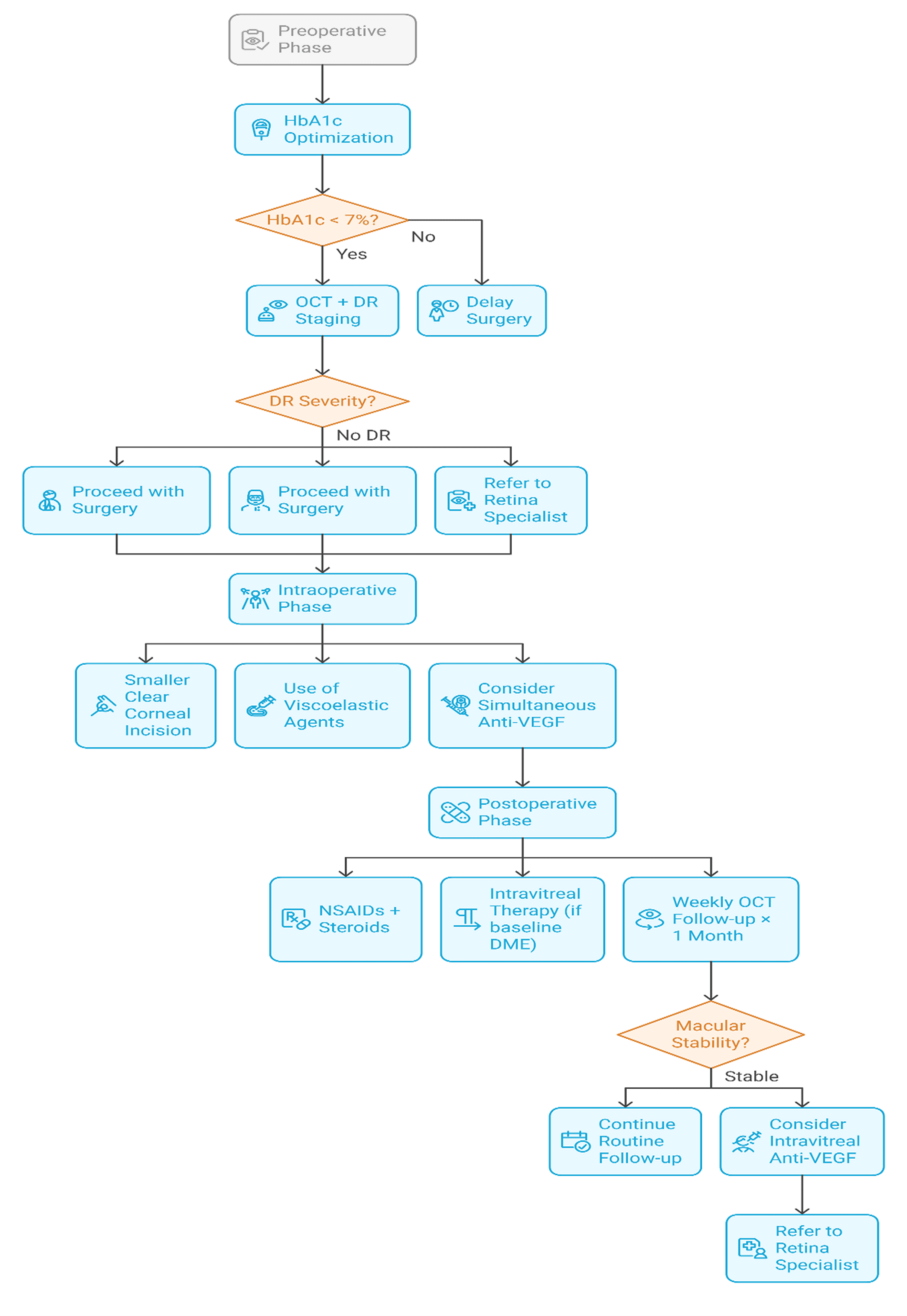
Workflow for Preoperative and Postoperative Management of Diabetic Patients Undergoing Phacoemulsification Created by authors HbA1c: glycated hemoglobin, OCT: optical coherence tomography, DR: diabetic retinopathy, VEGF: vascular endothelial growth factor, NSAIDs: nonsteroidal anti-inflammatory drugs, DME: diabetic macular edema

Secondly, there is significant heterogeneity in the ways of classification of DR among studies (e.g., grading inconsistency, variability in the inclusion of status of DME). Such fluctuation makes comparisons difficult and minimizes the possibility of generalizing research findings as a result of applying them to a clinical environment. Further, the absence of multicentric long-term prospective studies that measure visual prognosis past one year, specifically regarding systemic diabetic control and length of disease, is noteworthy. Such gaps make it necessary to conduct more rigorous and uniform research aimed at evidence-based care and surgical planning for diabetics after surgery. The implementation of a systematic approach to perioperative management of diabetic patients is described in Figure 3, providing a stepwise approach to getting the best results by multidisciplinary planning and early interventions.

Future research directions

Need for Large-Scale Prospective Studies

It is urgently needed to have large, multicentric, prospective studies, stratifying the outcomes according to glycemic control, duration of diabetes, and the severity of retinopathy. To perform meta-analyses and develop guidelines, these studies are to apply standardized surgical procedures and validated measures of visual functions. The connection between phacoemulsification and the course of diabetic eye disease will also be elucidated by longitudinal follow-up of 2-5 years [44,53].

Innovations in Imaging and Biomarkers

New modalities of imaging, like OCT angiography and ultra-widefield fluorescein angiography, have the potential to give information about subclinical ischemia, vascular density, and post-operative inflammatory changes [53]. The possible predictors of poor healing or the development of edema include biomarkers such as VEGF, IL-6, and tear film proteins [4,59]. The use of artificial intelligence in the process of imaging interpretation and outcome prediction has the potential to allow real-time risk scoring and decision support systems specific to diabetic patients [60].

Long-Term Visual Prognosis Studies in Different Diabetes Durations

Not much has been done regarding the effect of the duration of diabetes on long-term surgical outcomes [61]. There is an uncertainty as to whether long-standing, early-onset diabetes is more susceptible to varied profiles of complications than is late-onset or newly identified diabetes [62]. The visual prognosis in these subgroups in the long term needs to be further studied to determine the time to surgery and the approach to surgery [63].

## Conclusions

Phacoemulsification has become the standard of cataract extraction that provides good visual rehabilitation in any patient group. Nevertheless, in patients with diabetes mellitus, the situation is more complicated because of the change in ocular physiology, increased risks of complications, and unpredictable visual recovery. In this review, it has been mentioned that diabetic patients are more likely to face intraoperative complications like poor dilation of the pupil, fragility of the capsule, and postoperative complications like macular edema, increased inflammation, PCO, and vitreous traction on the retina and development of diabetic retinopathy. Although outcomes are generally good in diabetic patients, they are somewhat worse than in non-diabetic patients in the short term, especially where there is diabetic retinopathy or poor glycemic control. However, visual outcomes can be substantially enhanced with an optimized systemic and ocular management with preoperative screening, the perioperative anti-VEGF or corticosteroid drugs, and surgery personalization. Clinically, a multidisciplinary and patient-centered approach is essential. The stress should be put on glycemic optimization, realistic patient counseling, risk stratification, and strong postoperative monitoring, which helps to minimize the complications and enhance patient satisfaction. In the future, massive, prospective investigations and developments in imaging, biomarkers, and AI-guided decision-making instruments would play a critical role in the optimization of perioperative approaches. The provision of surgical care to diabetic patients according to their unique needs is not just a clinical necessity but a passport to a fair and successful ophthalmic performance.
